# Extrapolation chamber mounted on perspex for calibration of high energy photon and electron beams from a clinical linear accelerator

**DOI:** 10.4103/0971-6203.48718

**Published:** 2009

**Authors:** R. Ravichandran, J. P. Binukumar, S. S. Sivakumar, K. Krishnamurthy, C. A. Davis

**Affiliations:** Medical Physics Unit, Department of Radiotherapy, National Oncology Center, Royal Hospital, Muscat, Sultanate of Oman

**Keywords:** Calibration, extrapolation chamber, high energy beams, radiation standards

## Abstract

The objective of the present study is to establish radiation standards for absorbed doses, for clinical high energy linear accelerator beams. In the nonavailability of a cobalt-60 beam for arriving at Nd, water values for thimble chambers, we investigated the efficacy of perspex mounted extrapolation chamber (EC) used earlier for low energy x-rays and beta dosimetry. Extrapolation chamber with facility for achieving variable electrode separations 10.5mm to 0.5mm using micrometer screw was used for calibrations. Photon beams 6 MV and 15 MV and electron beams 6 MeV and 15 MeV from Varian Clinac linacs were calibrated. Absorbed Dose estimates to Perspex were converted into dose to solid water for comparison with FC 65 ionisation chamber measurements in water. Measurements made during the period December 2006 to June 2008 are considered for evaluation. Uncorrected ionization readings of EC for all the radiation beams over the entire period were within 2% showing the consistency of measurements. Absorbed doses estimated by EC were in good agreement with in-water calibrations within 2% for photons and electron beams. The present results suggest that extrapolation chambers can be considered as an independent measuring system for absorbed dose in addition to Farmer type ion chambers. In the absence of standard beam quality (Co-60 radiations as reference Quality for Nd,water) the possibility of keeping EC as Primary Standards for absorbed dose calibrations in high energy radiation beams from linacs should be explored. As there are neither Standard Laboratories nor SSDL available in our country, we look forward to keep EC as Local Standard for hospital chamber calibrations. We are also participating in the IAEA mailed TLD intercomparison programme for quality audit of existing status of radiation dosimetry in high energy linac beams. The performance of EC has to be confirmed with cobalt-60 beams by a separate study, as linacs are susceptible for minor variations in dose output on different days.

## Introduction

Calibration of photon and electron beams from a medical linear accelerator is carried out by using absorbed dose calibrated gas cavity chambers in water phantoms and applying different international protocols.[1–4] Accuracy of the measured doses must be within 2%, so as to achieve an overall delivery of radiation doses to patients within 5% accuracy limits, for the expected clinical outcome. The International Atomic Energy Agency (IAEA) network of secondary standard laboratories maintains national standards for traceability of field level dosimeters. Realization of absorbed doses in the primary standard laboratories is achieved from absolute measurements using, (a) standard graphite ion chambers (to determine absorbed dose to water) (b) graphite calorimeters in water, and (c) chemical dosimeters with Fricke solutions.[1]

For low-energy x-rays and beta radiations, Bohm and Schneider[5] developed an extrapolation chamber (EC), which is a specially designed parallel plate ionization chamber, capable of accurately measuring the differential specific charge (dq/dm), by varying air mass in the cavity by precise control of electrode separation. The principle and application of the EC for measurement of x-ray outputs have been reported in literature.[6–8] Zankowski and Podgorsak[9,10] reported the efficacy of specially built extrapolation chambers as an integral part of polystyrene and solid water phantom, to measure absorbed doses in cobalt-60 gamma beam, 4 to 18 MV x-rays, and 6 to 22 MeV electron beams. Mehenna Arib[11] reported their experience in performing absolute dosimetry with high-energy photon beams using a commercially available Perspex embedded EC and compared it with water measurements. If realization of the absorbed dose using these chambers is achieved from first principles, this chamber could become a departmental standard, in the absence of any traceable calibration, for the users.

In our institution we do not have a standard cobalt-60 machine for determination of N_d,water_ factors for thimble chambers. There is no secondary standards laboratory in this country for traceability of our beam level dosimeters. Therefore, keeping the objective of developing our own radiation standards for the absorbed dose, with high energy photons and electrons, we investigated the role of the EC for measurement of absorbed doses, with clinical radiotherapy beams. The doses measured with the EC were then compared with those estimated for water, using TRS 398 protocols with thimble ion chambers.

## Theory of Extrapolation Chambers

A brief account of the construction and working of an EC for measurement of the absorbed dose is given by Kron.[12] The dose estimated by air cavity in a medium D_med_ (Spencer-Attix cavity relation) is given by Eq. (1).

(1) $${\text{D}}_{\text{med}}=\left(\text{Q/m}\right).{\text{W}}_{\text{air}}.{\text{S}}_{\text{med.air}}$$

Where Q is the charge collected under saturation condition in the chamber sensitive air mass ‘m’, W_air_ = 33.97eV is the mean energy required to produce an ion pair in air, and S_med.air_ is the ratio of the restricted collision mass stopping powers of the medium and air for the electron spectrum, at the position of the cavity. The sensitive air mass satisfies the Bragg-Gray cavity condition, which stipulates that the cavity is so small that its presence does not perturb the charged particle field in the medium.

For small air cavities, the ratio Q/m is a constant, allowing Q/m to be replaced by the easier-to-measure derivative dQ/dm. The modified Spencer-Attix relationship of the dose with the medium is given by the relation

(2) $${\text{D}}_{\text{med}}=\left(\text{dQ/dm}\right).{\text{W}}_{\text{air}}.{\text{S}}_{\text{med.air}}$$

For parallel plate ionization chambers with variable air volume, Eq (2) can be rewritten as

(3) $${\text{D}}_{\text{med}}=\left(\text{1/}\rho Α\right).\left(\text{dQ/dz}\right){\text{.W}}_{\text{air}}.{\text{S}}_{\text{med.air}}$$

Where ‘ρ’ is the density of air volume, A is the effective area of the measuring electrode, ‘z’ is the separation between the polarizing and measuring electrodes. The perturbation caused by the air cavity in the medium is overcome by making the size infinitesimally small. This is achieved by the EC, making it possible to accurately measure the value of dQ/dz.

## Materials and Methods

### Radiation beams

There are two linear accelerators in the center, Clinac 2300 CD and Clinac 600 CD (Varian, USA). The available radiation beams are 6 MV, 15 MV X-Rays, and 6, 9, 12, 15, 18, 22 MeV electrons with 1m focus-axis-distance (FAD).

### Extrapolation chamber

A Perspex embedded EC Model 23392 (SI. No. 117, M/s PTW Freiberg, Germany) with the following specifications is used in this study (PTW Technical Documentation[13]). Chamber entrance window - 0.0035mm polyethylene terephtalate (PETP, Hostaphan) mylar foil. The area of the entrance window is 0.66 mg/cm^2^. The measuring volume can be varied between 0.353 to 7.422cm^3^ by moving the electrode using a piston operated by a micrometer screw. The distance between the electrodes is variable from 0.5mm to 10.5mm with the accuracy of parallelism being +1μm.

**Figure 1:**
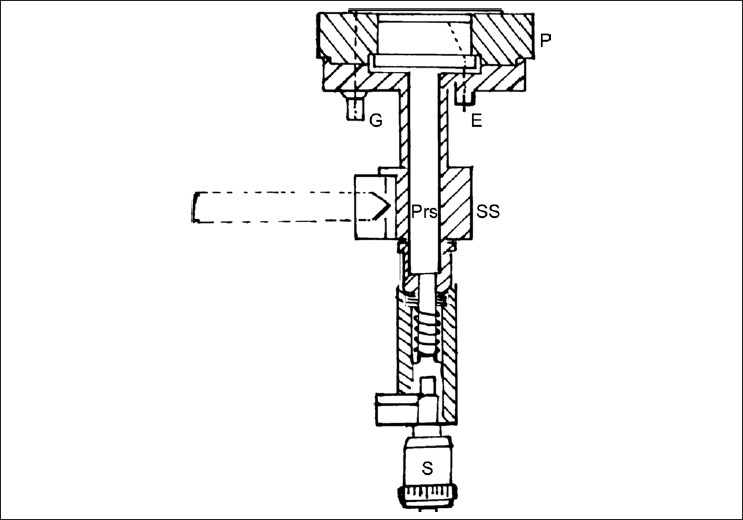
Schematic diagram of the extrapolation chamber (EC). G-Ground, E-Bias voltage to collecting electrode, SS-Stainless steel body, P-perspex body of EC, Pis-Piston, S-Micrometer screw

**Figure 2:**
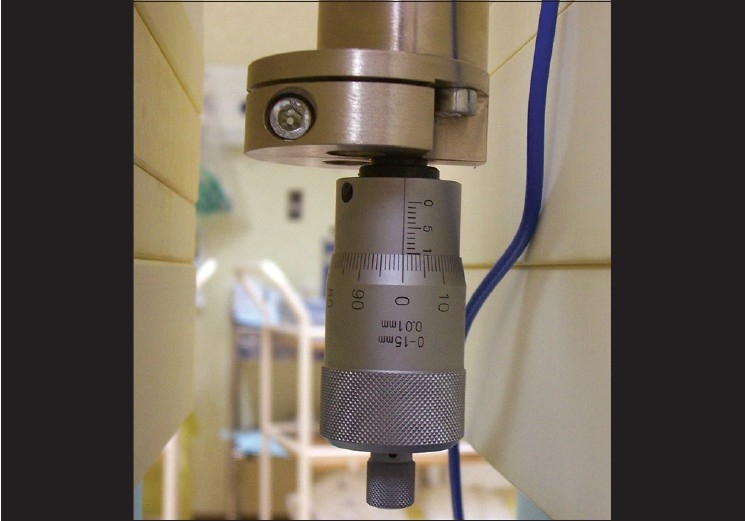
Micrometer design for selection of plate separation in the extrapolation chamber (EC)

Diameters of the entrance window and the rear electrode are 60.5mm each. The rear electrode is made up of perspex and methyl methacrylate (PMMA), with a graphite coated surface. The diameter of the measuring electrode is 30mm. There is a guard electrode with a width of 14.8mm and an insulating ring with a thickness of 0.2mm and a width of 0.2mm. The design of the chamber is such that the leakage current is < 10^−12^ A. For effective plate separation of 0.5mm, the saturation effect occurs at a voltage of >50V (99.5% saturation) for dose rates as high as 335Gy/s. Figure 1 and Figure 2 are the schematic views of the EC and the design of the micrometer screw arrangement.

### Electrometer

The chamber is connected to a Unidos electrometer (PTW, Freiburg) with dual polarity and available voltages up to 400V, in steps of 50V. The leakage of the ionization measurement is as low as 1 pC. The recombination correction is calculated by measurement of charges repeated with +400V and +200V, using the TRS 398 (IAEA)^1^ protocol. For electron absorbed dose measurements, the charges are averaged for +400V and −400V bias voltages.

### Geometry of measurement

**Figure 3:**
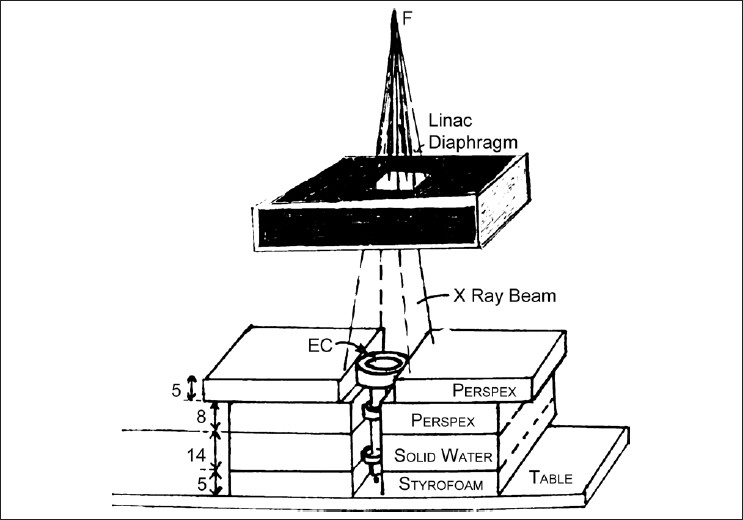
Geometry of calibration of the absorbed dose with extrapolation chamber

**Figure 4:**
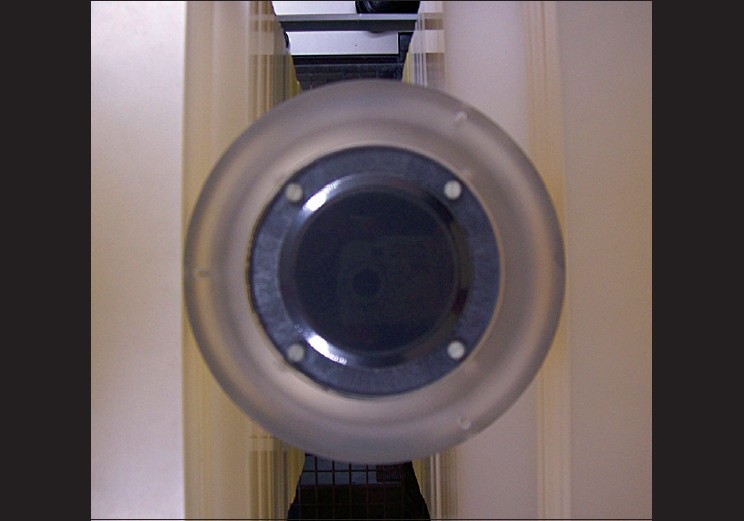
End on view of ion collection volume in EC. Outer perspex body (circular) and the perspex phantom underneath, during measurement, are seen in the figure

Figure 3 shows the geometry of measurement of the absorbed dose for photon and electron beams. Figure 4 shows the end on view in the direction of the beam entrance. The chamber wall is kept at an isocenter distance of 100cm for photons. The required thickness of 15mm and 28mm perspex, necessary to give the maximum buildup for a dose of 6MV and 15MV beams, respectively, are added on the surface level of the EC for measuring the absorbed dose/Mu for photons. For electron energies of 6 MeV and 15 MeV, the measurements are performed at 15mm and 35mm buildup depths by keeping the perspex plates on the surface of the entrance window of the EC and adjusting 100cm at the surface of the phantom. A field, 10cm × 10cm, is used at the isocenter for both photon and electron beams. The plate separation is decreased progressively from 10mm to 1.0mm by adjusting the micrometer screw. The ionization charge is collected for 100 MU exposures. The leakage charges are measured for each setting.

The charge in nC, corresponding to 1.5mm plate separation, is used for calculation of the absorbed dose (based on the recommendation of Zankowski and Podgorsak,[10] suggesting 1 to 2mm as the true Bragg-Gray region for the EC). Recombination correction is carried out by using the standard two voltage technique +400V and +200V. For electron beams, the charge collected for +400V and −400V are averaged. Measurements carried out during the period December 2006 to June 2008 have been analyzed. The calculated readings have been converted to absorbed dose in perspex by using Eq. (4). The values for ρ = 1.293 × 10^−3^ g/cm^3^ at standard pressure P_°_ = 1013 hPa, temperature T_°_ = 273.15K, and A = π. 0.00149^2^ sq.m, where the effective radius of the collecting volume is 14.9mm.

(4) $${\text{D}}_{\text{perspex}}=\left(1/\rho \text{A}\right).\left(\text{dQ/dz}\right).{\left(\text{W}\right)}_{\text{air}}{\text{.S}}_{\text{perspex}..\text{sw}}{\text{S}}_{\text{sw.air}}$$

Table 1 shows the values of stopping power ratios of perspex to solid water (SW) (Seuntjens *et al*.[14]), and solid water to air (Zankowski and Podgorsak[10]) used in Eq. (4) to derive the absorbed dose to water.

**Table 1:** Stopping power ratios used for absorbed dose calculations

*Beam quality*	*Energy*	*5_perspex..SW_*	*S_SW.air_*
X-Rays	6 MV	0.9919	1.106
electrons	15 MV	0.9914	1.071
	6 MeV	0.9900	1.075
	15 MeV	0.9900	1.013

Calculation of absorbed dose from EC readings.

The absorbed dose cGy/MU is calculated using the relation

(5) $${\text{D}}_{\text{perspex}}=33.87.\left(1/\rho \text{A}\right).\left(\text{dQ/dz}\right).{\text{S}}_{\text{perspex}..\text{sw}}{\text{S}}_{\text{sw.air.}}{\text{K}}_{\text{s}}.{\text{K}}_{\text{tp}}$$

For air with relative humidity of 45%, the value of W_air_ is 33.87eV and dQ/dz is obtained experimentally for each radiation beam, with appropriate buildup conditions. K_s_ and K_tp_ are corrections for recombinations and for air density variations with respect to standard ambient conditions (20° C and 1013 hPa). The multiplication values for obtaining absorbed dose Gy/C are constants for different radiation qualities used, and are obtained by referring to appropriate conversion factors as in Eq. (5).

### Estimation of absorbed dose from thimble chambers

The reference absorbed dose data are made available by 0.6 cc (FC65) ion-chamber measurements in water, using the TRS 398 protocol in the Blue Phantom radiation field analyzer (Scanditronix Wellhofer). The ion chambers used in this study have Nd water calibration factors supplied from Scanditronix Wellhofer Laboratories (Germany). The 0.6 cc chamber is connected to Dose 1 reference standard electrometers. Absorbed doses in the water were obtained on the same day when EC measurements were carried out, to account for the corresponding radiation outputs in linear accelerators.

## Results

### Measured absorbed doses with EC

**Figure 5:**
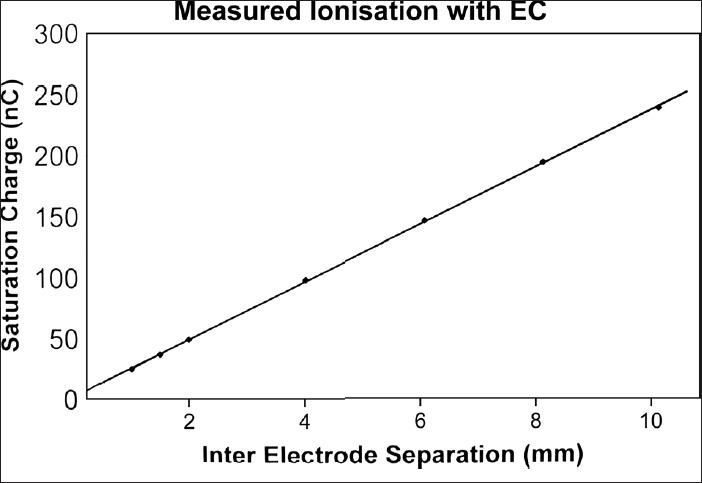
Variation in the uncorrected ionization charge (nC) from EC, for 6 MV x-rays, for different inter-electrode separations

Figure 5 shows the representative variation of the collected ionization charges in nC, for various plate separations, obtained using micrometer screw adjustments. Tables 2 and 3 show the summary of results from many measurements of photon and electron beams, using the EC, obtained over a long period of time. The mean values of each plate separation, without applying other corrections, along with the standard deviations are shown. It can be seen that over a long period of time, all the measurements are within a maximum standard deviation of 2% despite variations in ambient conditions.

**Table 2:** Measured uncorrected ionization charge for high-energy X-ray beams

*Measured period*	*X-ray energy/Machine*	*Collected mean charge (nC/100MU) for various inter-electrode separations (mm) (micrometer settings)*						
		
		*10*	*8*	*6*	*4*	*2*	*1.5*	*1.0*
Dec/06 to May/07	6MV CLINAC	226.1±	184.0±	140.0±	94.7±	48.4±	36.7±	25.05±
n = 3	600CD	0.64%	0.57%	0.45%	0.33%	0.03%	0.64%	0.20%
Jul/07 to June/08	6MV CLINAC	235.4±	191.4±	145.4±	98.5 ±	50.6±	38.4±	26.3±
n = 6	2300 CD	0.93%	1.05%	1.12%	1.23%	1.32%	1.42%	1.70%
Jul/07 to June/08	15MV CLINAC	235.8±	193.9±	147.9±	100.5±	51.7±	39.3±	26.8±
n = 6	2300 CD	1.64%	1.89%	1.76%	1.86%	1.88%	1.93%	1.94%

**Table 3:** Measured uncorrected ionization charge for high-energy electron beams

*Measured period*	*Electron energy/Machine*	*Collected mean charge (nC/100MU) for various inter-electrode separations (mm) (micrometer settings)*						
		
		*10*	*8*	*6*	*4*	*2*	*1.5*	*1.0*
Jul/07 to June/08	6MeV CLINAC	226.2±	188.2±	145.7±	99.6±	51.0±	38.5±	26.2±
n = 3	2300CD	1.15%	1.35%	1.48%	1.72%	1.82%	1.96%	1.11%
Jul/07 to June/08	15MeV CLINAC	203.0±	170.2±	132.2±	90.4±	45.6±	34.3±	22.8±
n = 2	2300 CD	0.50%	0.61%	0.64%	0.74%	0.93%	0.76%	0.95%

Table 4 shows the calculated absorbed doses in the perspex, using the EC, compared with 0.6 cc ion chamber measurements in water. All the results of the EC-estimated absorbed doses show good agreement (not exceeding 2%) with well-established thimble chamber measurements.

**Table 4:** Absorbed doses measured with EC and comparison with thimble chamber data

*No.*	*Machine and beam*	*FAD/FSD Buildup thickness*	*Calibration factor(EC) cGy/nC*	*Recombination corr. Ks*	*Temp.press. corr. Ktp*	*dQ/dznC/mm*	*Measured absorbed dose by EC and 0.6cc chamber cGy/MU*		
							
							*EC*	*0.6cc*	*Devn. %*
1	CLINAC	100 cm	4.11776	1.0045	1.015	0.2483	1.0421	1.0447	−0.25
2	600CD	FAD			1.013	0.2489	1.0428	1.0447	−0.20
3	6MV	1.5 mm	4.11776	1.0045	1.016	0.2452	1.0297	1.0166	+1.3
1	CLINAC	Ppx BU			1.002	0.2608	1.0809	1.0622	+1.5
2	2300CD	100 cm			1.016	0.2570	1.0797	1.0572	+2.1
3	6MV	FAD	4.01521	1.010	1.011	0.2525	1.0554	1.0588	−0.3
4		1.5 mm			1.014	0.2576	1.0765	1.0759	+0.1
5	CLINAC	Ppx BU			1.021	0.2576	1.0933	1.0730	+0.7
6	2300CD		3.99468	1.0043	1.021	0.2559	1.0805	1.0599	+1.2
1	15MV	100 cm			1.002	0.2640	1.0728	1.0652	−1.7
2		FAD	3.76468	1.0143	1.016	0.2653	1.0927	1.0768	+1.1
3	CLINAC	2.8 mm			1.011	0.2555	1.0471	1.0744	+1.1
4	2300CD	Ppx BU			1.014	0.2657	1.0887	0.9909	+1.0
5	6MeV				1.021	0.2641	1.0983		
6	Electron	100 cm			1.021	0.2623	1.0858	1.0585	−0.85
1	CLINAC	FSD			1.010	0.2438	1.0345	1.0594	+0.2
2	2300CD	1.5 mm			1.021	0.2408	1.0013	1.0594	−0.30
	15 MeV	Ppx BU							
	Electron	100 cm			1.011	0.2680	1.0496		
1		FSD			1.010	0.2709	1.0610		
2		3.5 mm			1.021	0.2670	1.0566		
3		Ppx BU							

## Discussion

The present study has outlined the efficacy of calibrating the absorbed dose in high-energy linear accelerator radiation beams using an EC. This is possible from the first principles, because of the precise definition of the circular cavity area and the separation between electrodes is known to a high resolution of (1/100)th of a millimeter. The precise estimation of the ionization gradient (dQ/dz) is possible with the design feature of a Bohm-type beta standard ionization chamber (Bohm and Schneider[5]).

Earlier reports by Klevenhagen[8] and Zankowski and Podgorsak[9,10] have brought out the possibility of using such designed prototype versions of extrapolation chambers for the estimation of the absorbed dose from clinical linacs and tele-cobalt beams. These authors have used polystyrene and solid water phantoms for the construction of extrapolation chambers, and measured absorbed doses at selected depths in tissue-equivalent media. We have used the commercially available EC, in which the outer body is perspex and the inner piston is perspex, carrying an electrically conductive coating. In the above circumstances, we added the calculated stopping power ratio of perspex to solid water. Seuntjens *et al*.[14] have extensively discussed methods to determine absorbed dose to water from solid phantom measurements. We compared various phantoms and provided correction factors for deriving absorbed doses. For beam qualities 6MV and 15MV, the referred correction factors for stopping power ratio perspex to solid water are 0.9919 (1.088/1.097 = 0.9919) and 0.9914 (1.063/1.0723 = 0.9914), respectively. However, we have used these values to derive the absorbed dose calibration factor in Eq. (4). It can be seen that by multiplying the stopping power ratio of Perspex to solid water, then by ratio of solid water to air, we ultimately arrive at a dose to the Perspex material of the EC. Good agreement is seen with the absorbed dose in water [Table 4]. Therefore, it appears that some discrepancies arising due to nonwater equivalence of the EC material get canceled on account of, (a) absence of scatter owing to nonexistence of phantom material under the EC body [Figure 3] or (b) due to the presence of stainless steel metallic parts of the measuring system replacing the gap between the solid measuring phantom plates.

The diameter of the measuring electrode is 30mm, the guard electrode has a 14.8mm width, an insulating ring thickness of 0.2mm, and width of 0.2mm. We have used 14.9 (14.8 + 0.2/2)mm for effective radius, for calculating the area (A). In a recent report[11] have confirmed by measurements the effective diameter 30.04mm, which is in agreement with the quoted value from the technical document, from PTW-Freiburg. They have calibrated the EC to a standard cobalt-60 beam and used the EC for determination of the absorbed dose to high-energy photon beams. In our present study we have calculated the calibration factor for EC for different energies and used these factors [column 4, Table 4] to estimate the absorbed doses in high-energy beams.

The outer body surrounding the back side of the perspex piston and mounting of the micrometer screw arrangement are made of stainless steel. Its interference appears to be insignificant, though its influence on the back scatter has to be separately characterized. We feel that as the collecting volume is just below the mylar foil, the probability of backscattered photon will be insignificant. In Figure 5 the differential ionization charge has linear variation with inter-electrode distance ‘z’. The earlier work by Zankowski and Podgorsak (1997), has shown that the Bragg Gray cavity region holds good for ‘z’ values 1 to 2mm. In our work also, the value of (dQ/dz)_1.5cm_ has yielded a correct estimation of the absorbed doses. Moreover, it was observed from our data that substitution of dQ/dz values obtained from other plate separations underestimate the absorbed doses for all the radiation beams.

The use of the EC as radiation standards for absorbed doses, for clinical high-energy linear accelerator beams, is highly dependent upon precision machining of the chamber. In particular warping of perspex can occur over a period of time. This can significantly affect the volume and the extrapolated volume, particularly for the determination of zero volume data. The thimble chambers, generally used in the radiation physics departments, have fixed volumes, good stability, and reproducibility for a long time. In comparison, therefore, it may be necessary to have a standard graphite chamber as a parallel radiation standard to check the performance of the EC at fixed intervals.

Uncorrected ionization readings have not shown many variations on measurements carried out over many occasions, because the corrections for temperature and pressure were within 2% against a reference temperature of 20°C. In Table 4, the calibration factors for various beam qualities derived from Eq. (5) are listed for use with similar extrapolation chambers. Our initial experience with the EC shows that this will constitute one of the radiation standards in beam level dosimetry, to recognize the necessary accreditation in the area of radiological standards. The same measurements may have to be repeated in ^60^Co beams, for confirming the application of EC for such beams. However, because of the nonavailability of ^60^Co machines around our institution we could not perform such a study.
